# Annotations

**Published:** 1897-07-24

**Authors:** 


### Annotations.

### Parks and Poor Rates.

One of the curious results of our system of local self-government is the tendency shown by public bodies to levy rates upon one another. Some time ago Brockwell Park was handed over to the London County Council, being vested in them solely for the purpose of maintaining it for the free use of the public. This, one might have thought, was a distinct benefit to the district in which the park lay, and it did not seem unreasonable to look upon that district as in a sense beholden to the County Council for maintaining the park at the expense of the general metropolitan rates. To the poor-law guardians, however, the matter appeared in quite a different light. Here is a park, they seem to have said, which must be owned by someone or other, and whoever owns it must pay the poor rate. So the good people of Lambeth, having got the park, and having thrown the support of it upon the County Council, calmly turn round and ask this body also to take a part in the payment of the local poor rate. Perhaps even more curious than the claim was the fact that it was supported by a decision of the Court of Queen's Bench. This, however, was reversed by the Court of Appeal, and the reversal has now been maintained on a further appeal being made to the House of Lords. There is a certain reasonableness in parks and open spaces being maintained at the general expense for the benefit of the poorer districts. It is quite otherwise, however, when the recipients of these favours demand that these parks should be rated for the support of the local poor.

### The British Medical Association and its Helpless Members.

We regret to hear that the British Medical Association has removed from its ranks the two gentlemen who accepted posts at the Adelaide Hospital in opposition to the wishes of a majority of the members of the South Australian branch. The Government of South Australia was represented by counsel at the meeting of the Council of the Association at which this decision was arrived at, and no doubt everything that was possible was done to save the Association from so ill-judged a proceeding. We understand that the British Medical Association has, by its constitution, absolute power to expel any member without reason given, and we presume that it feels assured that in the course it has adopted it has acted in accordance with the law. We do not, however, feel quite clear that no remedy will lie against them for damage done. What we are quite certain of is that in accepting the membership no man understands or admits that he is liable to be expelled at the sweet will of the Council. There is always an implied condition, namely, that this power of expulsion shall not be exercised except for just and grave reasons, such, for example, as would justify an accusation of conduct "infamous in a professional respect." Now it is obvious that the persons concerned in this matter have not been guilty of any such conduct. They have merely offended the medical men in Adelaide by accepting posts which they had declined. This is not a matter of under-selling one's fellows, it is not a matter of medical ethics, of interfering with other men's patients, or of doing anything contrary to the general customs of the profession. It is merely that these men accepted posts which the Adelaide doctors had thrown up; and the reasons for their throwing them up were not that they had been asked to do anything unprofessional, but merely that they were dissatisfied with the arrangements made for the governance of the hospital. We cannot for a moment admit that such a thing is a just cause for attaching to any man the grave stigma which must be cast upon him by expulsion from a society of gentlemen such as the British Medical Association; and we cannot but see that if it should happen that those who have been expelled should endeavour to recover damages for the injury which they must undoubtedly have suffered, the governing body of the association will find themselves in a somewhat awkward predicament. The more they maintain the rectitude and honesty of the association, and the advantages of belonging to it, the greater will be the proof of injury done by expulsion from its ranks; while, if they try to lessen the damages they must pay, they will have to cry "stale fish" about the membership, and all its boasted advantages—a curious dilemma. If, however, it is gravely maintained that the Council has the right to expel any person, it is clear that to become a member of the British Medical Association, and thus to submit one's character to be the plaything of so irresponsible a tribunal, is a somewhat serious matter, and one to be undertaken with much circumspection.

### The Adulteration of Food.

Is it not time that we should make an end of the ridiculous arrangements under which samples of foods and drugs are now obtained for analysis by the authorities? The inspectors are in most cases perfectly well known, and where strangers are employed not only do they often turn out very unreliable witnesses, but there is something ignominious and lowering to the dignity of the law in the whole performance. The difficulty arises from the fact that the sample must be bought, and that the purchaser must take what is given to him, so that it is quite possible, where the inspector is known to the shopman, for the latter to serve him with an unadulterated article, while in the very next drawer there may lie the adulterated stuff which he habitually furnishes to his unsuspecting customers. In this particular point the Italian sanitary law is far ahead of ours. In that country the medical officer of health, or other officer appointed by law, may enter a shop and inspect the whole of the stock, and may take away with him any sample he pleases. It is, in fact, taken for granted that what is in a shop is there with the intention of being sold. The principle of open seizure is already sanctioned by law in regard, for example, to milk in transit, and, seeing that the law prohibits the sale of adulterated food, it does not seem unreasonable that the storing of such stuff in a shop opened for the sale of food should be taken as showing an intention to break the law. At present, so far as concerns the retail trader, the actual sale must be proved, which is often far from being a simple matter, whereas it would be the simplest thing in the world to look in the grocer's cupboard. But liberty, even the liberty of the retail grocer, is a holy thing with which no inspector may interfere.

